# Therapeutic Management of Locally Advanced Rectal Cancer: Existing and Prospective Approaches

**DOI:** 10.3390/jcm14030912

**Published:** 2025-01-30

**Authors:** Horia-Dan Lișcu, Nicolae Verga, Dimitrie-Ionuț Atasiei, Andreea-Teodora Ilie, Maria Vrabie, Laura Roșu, Alexandra Poștaru, Stefania Glăvan, Adriana Lucaș, Maria Dinulescu, Andreea Delea, Andreea-Iuliana Ionescu

**Affiliations:** 1 Department of Oncological Radiotherapy and Medical Imaging, “Carol Davila” University of Medicine and Pharmacy, 050474 Bucharest, Romania; horia-dan.liscu@drd.umfcd.ro (H.-D.L.); nicolae.verga@umfcd.ro (N.V.); teodora.i.ilie@stud.umfcd.ro (A.-T.I.); maria.p.vrabie@stud.umfcd.ro (M.V.); laura.rosu@stud.umfcd.ro (L.R.); alexandra.postaru@stud.umfcd.ro (A.P.); adriana.lucas@stud.umfcd.ro (A.L.); maria.dinulescu@stud.umfcd.ro (M.D.); andreea-iuliana.miron@drd.umfcd.ro (A.-I.I.); 2 Radiotherapy Department, Colțea Clinical Hospital, 030167 Bucharest, Romania; andrreeadelea@gmail.com; 3 Department of Medical Oncology, Colțea Clinical Hospital, 030167 Bucharest, Romania

**Keywords:** locally advanced rectal cancer, treatment, TNT, neoadjuvant therapy, short-course radiotherapy, long-course radiotherapy

## Abstract

Rectal cancer (RC) presents significant challenges in diagnosis and treatment, with increasing incidence among younger populations. Treatment approaches, particularly for locally advanced rectal cancer (LARC), have evolved, notably with the introduction of total neoadjuvant therapy (TNT). TNT combines neoadjuvant chemotherapy and chemoradiotherapy before surgery, improving overall survival and reducing both metastasis and local recurrence rates compared to traditional methods, while enabling more patients to complete the full oncological treatment. Clinical trials, such as RAPIDO, OPRA, and PRODIGE 23, have demonstrated the effectiveness of TNT in tumor downstaging and complete pathological responses, offering better outcomes for patients; however, debates persist regarding the role of neoadjuvant radiotherapy, with novel strategies exploring its omission in specific cases to reduce toxicity and enhance quality of life. In addition, organ preservation strategies, such as the watch-and-wait (WW) approach, have emerged as viable options for patients with a complete response to neoadjuvant therapy. Future directions point towards personalized treatment plans incorporating radiogenomics and the integration of artificial intelligence into diagnostics to optimize patient outcomes. This review aims to synthesize current treatment strategies and ongoing advancements in rectal cancer management, providing insights into potential future innovations.

## 1. Introduction

In the new global, fast-paced environment, rectal cancer is a central issue of healthcare in terms of diagnosis, treatment, disease burden, and quality of life (QoL) [1]. The estimated number of deaths from 2020 to 2024 due to colorectal cancer (CRC) decreased in the US [2]; however, when stratified by rectal cancer (RC) solely, the cumulative number for both sexes increased by almost 6% in 2024, compared to the last 4 years (from 43,340 to 46,220) [3]. In terms of incidence and mortality, CRC is situated in the third position in the hierarchy, globally [4]. In the United States, the same pattern could be observed, with CRC being the fourth cause of mortality. Traditionally, RC has subscribed to the belief that an individual should do screening tests after the age of 50. While the overall incidence and mortality rates of RC could be declining, there has been a concerning rise in cases among younger adults, specifically those under 50 years of age, contrary to the classic evidence. This trend has been observed not only in the US but also globally. Moreover, the incidence rate of CRC in individuals under 55 has been increasing by 1% to 2% annually since the mid-1990s. Additionally, the mortality rate among young people has been rising by about 1% each year since the mid-2000s. By 2030, the incidence of colon cancer in this group is expected to rise by 90%, and that of RC by 124.2% [3].

In the majority of high-income countries, the standard of care involves neoadjuvant chemoradiotherapy (NCRT), followed by total mesorectal excision (TME) and subsequent adjuvant chemotherapy in patients with locally advanced rectal cancer (LARC); however, only about two-thirds of patients undergo the intended adjuvant chemotherapy (ADJCHT) due to complications related to the surgery or stoma, or because of patient preference [5]. A growing body of literature indicates the importance of total neoadjuvant therapy (TNT) in RC. While NCRT represents an essential pillar in the management of LARC, leading to tumor downstaging and the achievement of a pathological complete response (pCR), TNT has emerged as a promising strategy aimed at enhancing both local and systemic disease control. Although certain patients may opt for a non-surgical approach, TME continues to serve as the gold-standard intervention. While TNT demonstrates comparable efficacy to NCRT in terms of short-term oncological endpoints, limited data are available regarding perioperative outcomes [6]. Moreover, the role of neoadjuvant radiotherapy in the treatment of patients with LARC, who have undergone a priori neoadjuvant systemic therapy, has recently sparked considerable debate in the literature. This rationale is mainly based on recent clinical trials, including the Rectal Cancer and Preoperative Induction Therapy Followed by Dedicated Operation (RAPIDO), POLISH 2, and Neoadjuvant Chemotherapy with FOLFIRINOX and Preoperative Chemoradiotherapy for Patients with LARC (PRODIGE-23) clinical trials [7,8,9]. They have provided strong evidence supporting the efficacy of TNT—combining neoadjuvant chemotherapy and chemoradiotherapy before surgery—compared to traditional chemoradiotherapy approaches. The Chemoradiotherapy Plus Induction or Consolidation Chemotherapy as Total Neoadjuvant Therapy (CAO/ARO/AIO-12) and Organ Preservation of Rectal Adenocarcinoma (OPRA) trials further investigate the optimal sequence for administering neoadjuvant chemotherapy—whether as induction or consolidation—concerning long-course neoadjuvant chemoradiotherapy [10,11]. These studies have demonstrated favorable outcomes in terms of tumor downstaging, complete pathological response rates, and overall survival (OS). Moreover, TNT has shown potential benefits in terms of reducing treatment toxicity and preserving organ function, making it an attractive option for patients with LARC [12]. Overall, the results from these trials suggest that TNT represents a valuable therapeutic option that warrants consideration in the multidisciplinary management of LARC.

This study set out to investigate, in depth, the therapeutic options based on the recent findings in oncologic patients with RC, as well as the novelties that could reshape the future of how RC is managed.

## 2. Materials and Methods

For the literature review, in February 2024, a thorough literature search was carried out using PubMed, Google Scholar, and Scopus. The focus was on studies from the past five years (February 2019–February 2024), using keywords such as “locally advanced rectal cancer”, “treatment”, “epidemiology”, “diagnosis”, “neoadjuvant therapy”, “total neoadjuvant therapy”, “dose escalation”, “watch-and-wait”, “immunotherapy”, “proton-beam”, “hyperthermia”, and “MRI-guided radiation”. Additional references were extracted from the articles retrieved during the search. Inclusion criteria were studies published within the past five years, articles with abstracts and manuscripts written in English, and research that specifically addressed locally advanced rectal cancer. Exclusion criteria were studies published before February 2019, articles with abstracts and manuscripts not available or not in English, and research that did not specifically address locally advanced rectal cancer or related treatments. Since this was not a systematic review, the PRISMA flow chart, protocol heterogeneity, and selection bias charts were not applied.

## 3. Relevant Background of Rectal Cancer

The vast majority of RC is sporadic (70%), and is diagnosed at an average age of 50 [13]. A small number of patients (10%) have a hereditary risk for developing cancer when under 50 years old, while a small percentage have a familial variant present despite the lack of a hereditary syndrome. Approximately 5% of colon and rectal cancers are attributed to genetic disorders such as familial adenomatous polyposis and Lynch syndrome. In the United States, a difference in incidence for Caucasians, African Americans, Asians, and Hispanics has also been noted. The highest incidence is present in African Americans, followed by Caucasians and Hispanics. African American men have the highest mortality, while Asian and Hispanic women have the lowest. There are also differences in the stages of disease discovery, with African Americans presenting with local and regionally advanced rectal cancers less often than Caucasians, and with metastases present more often than in Caucasians, in which cases the disease is often fatal [14,15].

In the context of LARC, defined as stage II (T3-4, lymph nodes negative) or stage III (lymph nodes positive), the 5-year survival rate is between 69 and 87% due to improvements in several diagnostic and treatment approaches, such as TNT, MRI, rectal echoendoscopy, radiotherapy, and TME [16]. Local recurrence is linked to higher mortality and is attributed to surgical failure with negative resection margins, locations, and advanced stages of the primary tumor; however, the recurrence rate decreased following the introduction of TME, the identification of resection margins, and neoadjuvant radiotherapeutic treatment followed by surgery. In the early 1920s the recurrence was around 20–30%; nowadays, the recurrence rate has diminished to 4–8% [17].

**Figure 1:**
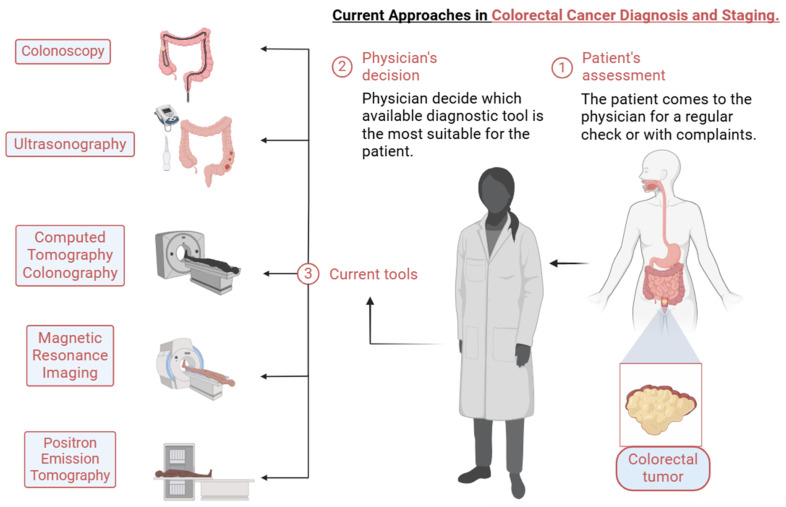
Current approaches in colorectal cancer diagnosis and staging that aid in treatment decisions. Firstly, the patient visits the physician for his annual check or gastrointestinal complaints. Being at risk of a colorectal tumor, the physician evaluates the patient, and, based on the patient’s medical history and clinical signs and symptoms, decides which technique is the most suitable. Several investigations are used for diagnosis (colonoscopy and computed tomography colonography) and complete staging (ultrasonography, magnetic resonance imaging, and positron emission tomography—in case of suspected or proven distant disease).

The reduction in the recurrence rate was achieved by tumor staging systems that help to determine prognoses, survival rates, the international standardization of case assessment, and treatment decisions. The current version used is the 8th edition developed by the American Joint Committee of Cancer implemented in 2018. Briefly, the management of RC before the treatment involves diagnostic techniques, followed by staging tools if needed. Several procedures are used for the description of the primary tumor, as well as the overall oncologic workup: (1) colonoscopy, (2) ultrasonography, (3) CT colonography, (4) magnetic resonance imaging, and (5) positron emission tomography; therefore, imaging plays an important role in choosing treatment strategies for patients with RC (Figure 1). In the following paragraph, we will briefly discuss MRI and PET.

MRI compared to CT and ultrasonography has superior soft-tissue contrast, differentiates cancerous tissue arising from the muscularis propria of the rectum, and defines tumor invasion of the mesorectal fascia. MRI is capable of evaluating all lymph node stations in the pelvis and lower abdomen with a wider field of view. In addition to size, it demonstrates internal signal inhomogeneity, which is predictive of lymph node malignancy. Moreover, MRI shows venous enhancement, which is characteristic of extramural vascular invasion—a negative prognostic factor. The typical pelvic MRI for staging RC comprises multiplanar images that are 3 mm thin [18]. The utility of intravenous contrast for enhancing tumor or lymph node staging using MRI has been questioned. Nevertheless, other studies have suggested that evaluating abnormal lymph node contrast could hold diagnostic significance. MRI findings can also serve as indicators of neoadjuvant therapy. A definitive indicator for neoadjuvant therapy is tumors staged as cT3 and cT4. Additionally, if an MRI shows positive lymph nodes, malignant invasion, or tumors invading or about to invade the mesorectal fascia, these are indications for preoperative treatment [19].

PET examination has been proven to be useful in the evaluation of RC, assessing lesions previously undetected on other types of examinations; however, a PET exam is not mandatory, but may be considered as an option in selected cases of metastatic disease, but surgically curable. Metabolic examination allows for the complete preoperative staging and identification of occult metastatic disease. Following such an investigation, one-third of patients with LARC experienced a change in therapeutic management [20]. Furthermore, functional imaging, such as 18-fluorodeoxyglucose positron emission tomography (18-F-FDG-PET), can assess the tumor response to therapy. Some authors have found that 18F-FDG PET performance is comparable to histopathological examination in predicting the effectiveness of neoadjuvant therapy in treating patients with LARC [21]; however, given the relatively low resolution of PET scans (approximately 3–5 mm transaxially centrally located in the field of view), 18F-FDG-PET is unable to differentiate a significant response from a full tumor regression following TNM restaging. In contrast, evidence from small studies suggests that 18F-FDG-PET/CT is superior to CT scans alone for TNM staging. Consequently, up to 36% of patients with LARC may experience changes in management with 18F-FDG-PET/CT [21].

## 4. Updates in Rectal Cancer Therapy

The treatment of LARC is still a challenge for the medical field, given the anatomy of the area and the proximity of vital structures. Treatment options include both curative surgery and neoadjuvant therapy aimed at downstaging the tumor. Neoadjuvant therapy (NAT) includes a wide range of options, such as radiotherapy and chemotherapy alone, or in combination [22]; TNT is an intensified NAT strategy that always combines radiotherapy and chemotherapy, though the optimal sequencing and dosing remain under investigation. NAT for the treatment of RC has several applications: (1) local disease control by treating tumors locally and reducing the risk of local recurrence [23]; (2) before surgery to reduce the tumor size [24]; (3) improving the chances of preserving the anus by making it possible to perform conservative surgery [11]; (4) reducing the risk of metastasis [25]; and (5) assessing the response to treatment [26].

Radiological staging and discussions within a multidisciplinary team are essential in selecting patients for neoadjuvant therapy. Some patients have a complete response after radiotherapy without further surgery, but they should be enrolled in national follow-up programs; however, like any type of treatment, it also has disadvantages such as the risk of anorectal and genitourinary dysfunction, which negatively impacts patients’ QoL [27].

**Figure 2:**
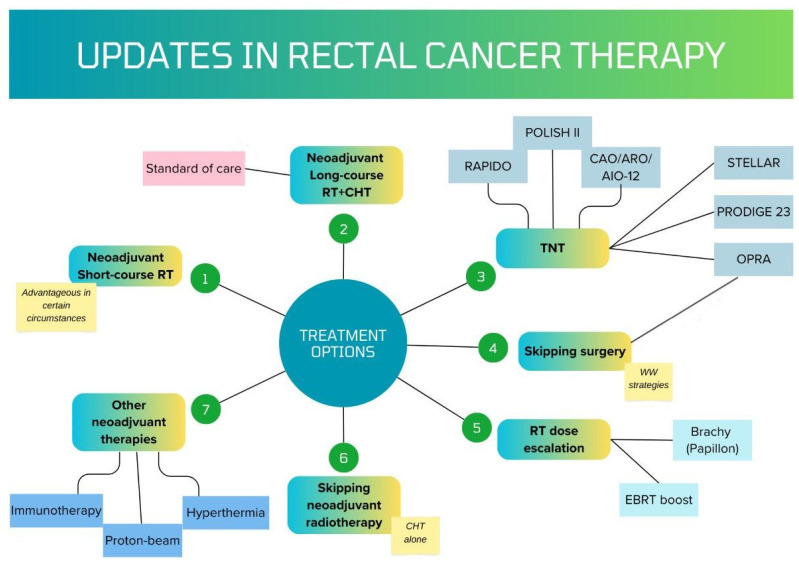
Main treatment options for locally advanced rectal cancer in 2024. Radiotherapy (RT); radiotherapy + chemotherapy (RT + CHT); total neoadjuvant therapy (TNT); Rectal Cancer and Preoperative Induction Therapy Followed by Dedicated Operation (RAPIDO) trial; Chemoradiotherapy Plus Induction or Consolidation Chemotherapy as Total Neoadjuvant Therapy (CAO/ARO/AIO-12) trial; short-term radiotherapy followed by chemotherapy versus long-term chemoradiotherapy in locally advanced rectal cancer (STELAR); Neoadjuvant Chemotherapy with FOLFIRINOX and Preoperative Chemoradiotherapy for Patients with LARC (PRODIGE 23); Organ Preservation of Rectal Adenocarcinoma (OPRA) study; watch-and-wait (WW); and external beam radiation therapy (EBRT).

For RC curative treatment, TME is considered the gold standard, defined by the complete removal of the rectum, together with the surrounding mesorectum, and lymphovascular fatty tissue (mesorectum); this approach has greatly diminished local recurrence rates. By combining surgical treatment with NAT, local control was maintained for a long period or even permanently. A summary of the actual therapies can be seen in Figure 2.

### 4.1. Neoadjuvant Short-Course Radiotherapy

Short-course radiotherapy (SCRT) consists of a dose of 2500 centi-Gray (cGy) divided into five equal doses/day usually 7 days before surgery. This approach has been demonstrated to reduce local recurrence in at least two phase III clinical trials [28,29]. The Swedish study noted first a 14% reduction in local recurrence in patients receiving surgical treatment combined with neoadjuvant radiotherapy [30]. Much of the evidence supporting the use of neoadjuvant radiotherapy comes from Kapitejin et al. [29]. This study demonstrates that the combination of radiotherapy given preoperatively and total mesorectal surgery was related to a decrease in local recurrence rates from 8.2% to 2.4% compared to surgery alone.

SCRT demonstrates high patient compliance with treatment and better tolerability due to reduced toxicity, making this approach an alternative treatment for patients with associated pathologies who cannot tolerate neoadjuvant radiochemotherapy. The short period between neoadjuvant radiotherapy and surgery has been shown to be in the patient’s favor, with a significantly reduced risk of tumor recurrence. As an example, the Swedish study has shown that preoperative radiotherapy with a dose of 25 Gy given one week before surgery for RC increases the survival rate and QoL of patients [30]; however, when the tumor invades the resection margins, NCRT is of choice, but in elderly patients with comorbidities NCRT can be a contraindication. SCRT in combination with surgery performed more than a week apart has been shown to be beneficial for these patients. Therefore, a delay of 6 to 8 weeks between neoadjuvant radiation and operation may be a viable option in elderly patients when preoperative SCRT is contraindicated [31].

The radiation dose is pivotal in defining the benefit of preoperative radiotherapy. Therefore, in Europe, studies that evaluated surgery alone versus neoadjuvant radiotherapy followed by surgery have been developed. Results showed a decrease in local recurrence between 50% and 60% if the radiation dose was moderately restricted; however, if the dose administered was low, the effects were minimal or absent. Thus, it has been found that preoperative radiotherapy is of choice in most European countries [32]. The response to neoadjuvant radiotherapy could be quantified by measuring certain biomarkers, such as special AT-rich sequence-binding protein-1 (SATB1), forkhead box protein K1 (FOXK1), FOXK2 protein, and focal adhesion kinase (FAK) protein. It is important that these biomarkers are examined and compared with each other, and the results should be related to pre-existing imaging and clinic–biological investigations. At present, no study in the literature has published data showing that the effect of neoadjuvant radiotherapy could be quantified by a biomarker, but their integration into clinical practice would be of major benefit in the treatment of RC [23,33].

### 4.2. Neoadjuvant Long-Course Radiochemotherapy

Long-course chemoradiotherapy (LCRT) consists of 4500–5040 centiGray (cGy) divided into 25–28 equal doses given over 5 to 6 weeks together with 5-Fluorouracil (5-FU) at a dose of 200 mg/m^2^ daily or capecitabine at a dose of 825 mg/m^2^ twice a day throughout the treatment period. Surgery is scheduled for 6 to 12 weeks after the completion of radiochemotherapy [34].

The period between the completion of LCRT and actual surgery has resulted in tumor regression, an important issue in the treatment approach for RC. The approach was adopted after various studies demonstrated the superiority of neoadjuvant LCRT over adjuvant LCRT, marked by the preservation of the anal sphincter and better local control of recurrences. Findings suggest that preoperative radiochemotherapy has provided substantial benefits in recent decades. Perioperatively, it has been associated with greater preservation rates of the anal sphincter and fewer local recurrences, thereby increasing survival [29].

Tumor location is a prognostic factor for treatment efficacy. Therefore, a randomized phase III clinical trial conducted by van Gijn et al. demonstrated that a rectal neoplasm located in the middle or lower level has a better outcome to NCRT than a superior localized RC [35]. The use of different NCRT regimens in patients with RC has also been considered. Thus, studies have been focused on giving radiotherapy in combination with capecitabine 5 days/week compared to giving radiotherapy with capecitabine and oxaliplatin. Nevertheless, short-term data revealed no notable changes in clinical outcomes between the two groups of patients [36]. Despite the documented advantages of neoadjuvant NCRT in terms of local disease control, patient survival, and the facilitation of anal sphincter-sparing surgery, uncertainty persists regarding the optimal timing of surgery. Francois et al. demonstrated that a prolonged interval of 6 to 8 weeks as opposed to 2 weeks between preoperative irradiation and surgery resulted in better tumor downstaging. In contrast, another study demonstrated a statistically significant increase in the response rate when surgery occurred more than 7 weeks post-irradiation. This highlighted the ongoing debate and the need for further investigation about the best timing for surgery after neoadjuvant radiochemotherapy [37].

Therefore, a randomized phase III clinical trial investigated the differences between neoadjuvant radiotherapy and NCRT. The results placed neoadjuvant radiochemotherapy on the beneficial side regarding the risk/benefit balance. LCRT has improved resection and local control, but increased toxicity in these patients; however, for late toxicity, there was no difference between the two types of treatment [30]. The current strategy of using neoadjuvant chemoradiotherapy followed by surgery and then adjuvant chemotherapy has a major drawback: a significant number of patients are unable to undergo adjuvant chemotherapy due to the side effects experienced after neoadjuvant chemoradiotherapy or, more notably, after major surgery. Adverse reactions such as radiation-induced proctitis, hematological imbalances caused by chemotherapy, or postoperative complications (e.g., infections, delayed healing, or low anterior resection syndrome) can have both physical and psychological impacts, discouraging patients from completing adjuvant chemotherapy. This challenge led to the development of the idea of administering chemotherapy in the neoadjuvant setting—TNT [38].

### 4.3. Total Neoadjuvant Therapy (TNT)

The therapeutic approach to LARC is continually evolving, supported by recent trial data on TNT. As previously stated, TNT combines neoadjuvant chemotherapy and chemoradiotherapy before surgery, improving overall survival and reducing metastasis rates compared to traditional methods, while enabling more patients to complete full oncological treatment. While current studies focus on determining the oncological effects of TNT, the lack of standardization in the protocols used by researchers makes the interpretation and comparison of data difficult. The interest in TNT is related to its effects on advanced cancers that may not have downstaging behavior similar to that of early cancers. TNT is the new trend in the approach to RC, to reduce distant metastases, improve local control, and preserve QoL. It involves the administration of cancer treatment chemotherapy (the sequencing will be discussed later), and chemoradiotherapy, in the preoperative setting. Its goal is to treat early infraclinical micrometastases, improve treatment compliance and ensure treatment efficacy, reduce time to ileostomy closure, and increase the primary tumor response.

The results from the Rectal Cancer and Preoperative Induction Therapy Followed by Dedicated Operation (RAPIDO) [7], POLISH-II [8], Chemoradiotherapy Plus Induction or Consolidation Chemotherapy as Total Neoadjuvant Therapy (CAO/ARO/AIO-12) [10], Organ Preservation of Rectal Adenocarcinoma (OPRA) [39], Neoadjuvant Chemotherapy with FOLFIRINOX and Preoperative Chemoradiotherapy for Patients with LARC (PRODIGE 23) [9,40], and STELLAR clinical trials [40] investigating TNT in the practical management of LARC are highlighted.

#### 4.3.1. RAPIDO

This study compared SCRT (25 Gy) followed by FOLFOX4/CAPEOX for 18 weeks with conventional LCRT (50 or 50.4 Gy, D/fr 2 Gy or 1.8, at the clinician’s discretion). The included group of patients had any of the following characteristics: T4, N2, EMVI, positive MRF involvements, or positive lateral lymph nodes.

The primary endpoint of this study was disease-related treatment failure (DrTF) at 3 years, defined as either a new primary colorectal tumor, the first occurrence of locoregional failure, distant metastasis, or treatment-related death. The patient group was divided into two arms: In the experimental arm, the approach was 5 Gy × 5 short-course radiotherapy (one hypofractionation with DT = 25 Gy), followed by six cycles of CAPEOX (capecitabine 1000 mg/m^2^, 2×/day, and oxaliplatin 130 mg/m^2^) or nine cycles of FOLFOX4 (oxaliplatin 85 mg/m^2^, leucovorin 200 mg/m^2^, bolus fluorouracil 400 mg/m^2^, and fluorouracil 600 mg/m^2^), followed by surgery [7]. In the conventional arm, the standard approach of 50 or 50.4 Gy (2 Gyfr or 1.8 Gy/fr) concomitant with capecitabine (825 mg/m^2^ ×2/day), followed by surgery, was maintained. Some patients then received ADJCHT with eight cycles of CAPEOX or twelve cycles of FOLFOX. In the experimental arm, DrTF was 23.7%, compared with 30.4% in the conventional arm. A significant improvement was also seen in terms of pCR; the results showed a pCR rate of almost double in the TNT group (28.4% vs. 14.3%), even though the experimental group used short-course radiotherapy. Tolerance to chemotherapy was approximately 85% in the TNT group and 90% in the LCRT group, who also received preoperative chemotherapy. Moreover, around 92% of patients in the experimental arm underwent surgery, compared with 89% in the standard group [7]; however, the 5-year follow-up study showed that the local recurrence rate was higher in the experimental arm compared to the conventional arm (10% versus 6%, *p*-value < 0.027) [41].

RAPIDO was the first study whose 3-year data markedly improved the DFS compared to standard therapy and showed that the TNT approach is an alternative for the management of LARC; however, fresh 5-year data show that local and regional recurrence rates are higher in the TNT group (10% vs. 6%), although they maintain a low rate of distant metastases.

The RAPIDO study summary is illustrated in Table 1.

#### 4.3.2. POLISH II

POLISH II trial was initiated in 2016, and the inclusion criteria were LARC—cT3, cT4, lymph node extension, without metastases; the research was performed on a group of 515 patients, and the options were SCRT (5 × 5 Gy) followed by 3 cycles of FOLFOX4 consolidation chemotherapy (n = 261), or LCRT (50.4 Gy + 5-FU, leucovorin + oxaliplatin—optional) (n = 254). Patients in both arms underwent surgery.

The data from this trial failed to achieve the objective of improving resection margins, as the two arms did not show a significant difference (77% in the experimental group compared to 71% in the standard group); there were minimal differences in complications and toxicities, but pCR was 16% in the TNT arm compared to 12% in the long-course arm; and no significant difference in pCR rates was observed. Additionally, OS at 3 years was significantly improved, 73% in the TNT arm compared to 65% in the conventional arm; however, data at 8 years show that OS is 49% in both arms. Therefore, a minor difference has been found between the standard and experimental groups in terms of DFS, namely 41% vs. 43% [8]. POLISH II is a trial that showed FOLFOX chemotherapy in only three cycles is not sufficient to bring improvement in DFS. It is noteworthy that this trial did not include MRI for comprehensive neoadjuvant therapy, which could potentially yield benefits and contribute to the improved management of LARC.

The POLISH II study summary is illustrated in Table 2.

#### 4.3.3. CAO/ARO/AIO-12

This is a study that was developed to compare induction chemotherapy (FOLFOX6) followed by LCRT (50.4 Gy, 1.8 Gy/fr for 28 days, in combination with 5-FU 250 mg/m^2^ and oxaliplatin 50 mg/m^2^) with chemoradiotherapy followed by consolidation chemotherapy [42].

Inclusion criteria were patients with locally advanced T3 rectal adenocarcinoma found in the distal or mid-rectum and T4 or lymph node involvement, assessed by MRI. A superior clinical pathological response is noted in the group that received consolidation chemotherapy, but the 3-year data indicate a rate of approximately 73% DFS in both groups; local relapses and distant metastases are similar in both arms of the study. Long-term oncological outcomes are not significantly different in the two groups, showing that this is a safe approach.

#### 4.3.4. OPRA

The OPRA study was impactful, even though it failed to achieve its objectives. The idea of increasing the tumor response with radiotherapy and its timing has been a debated topic over the last few years. This has revolved around the idea of chemoradiotherapy, then chemotherapy, followed by a watch-and-wait (WW) approach to follow tumor regression (if any). The rationale was that cancer treatment should be guided by the tumor response. The structure of the study was based on the differentiation of groups according to the sequence of chemotherapy administration, the intention being to preserve the rectum. Thus, the group was divided into one arm that received induction chemotherapy (FOLFOX × 8/CAPEOX for 16–18 weeks) and then underwent a staging evaluation (colonoscopy, MRI). The next step was chemoradiotherapy administration for 5.5 weeks (50.4 Gy, 1.8 Gy/fr) (INCT-NCRT group). The other group initially received chemoradiotherapy and underwent a consolidation chemotherapy regimen (NCRT-CNCT group). Both arms have been restaged using the “Memorial Sloan Kettering regression scheme”. If no clinically significant response was observed, they were referred for surgery (TME); however, if there was a clinically significant response, they were moved to the WW approach. Participants had to be over 18 years old, with clinical stage II (T3-4, N0) or stage III (any T, N1-2), a biopsy-confirmed rectal adenocarcinoma, and clinical staging using MRI, complete colonoscopy, and CT of the chest, abdomen, and pelvis [43].

The 3-year organ preservation results showed that the rates in the group receiving consolidation chemotherapy were 53% compared to the group receiving induction, which achieved 41%. At the baseline, the predictive values diminished from 35% to 25%, a significant change because about half of the patients managed to preserve the rectum without much change in the oncological outcome. Local regrowth rates were higher in the induction arm (40% vs. 27%), and DFS rates were similar in patients who had restaging surgery compared to those who had surgery for recurrence [43].

The 5-year results revealed that TME-free survival was 39% in the induction chemotherapy + chemoradiotherapy group and 54% in the consolidation chemoradiotherapy/chemotherapy group. At restaging (which took place approximately 8 weeks after TNT), approximately 28% of patients in the induction chemotherapy group underwent surgery compared to 24% in the consolidation group.

For patients in whom the WW approach was adopted, a local recurrence rate of 44% is observed in the induction chemotherapy/chemoradiotherapy group, compared to 29% in the consolidation chemotherapy/chemoradiotherapy group. In terms of DFS, the rates in the induction group are similar to those in the consolidation group, 71% vs. 69% [39]. cCR was observed in 125 (41.4%) patients and near-cCR in 104 patients (37.5%) out of the cohort of 304 analyzable patients, according to a secondary analysis published in 2024 [44].

Thus, it can be concluded that the response of rectal tumor formation to TNT is much higher than previously thought. Thus, a therapeutic strategy including TNT and a WW approach facilitates organ preservation in more than 50% of patients.

The OPRA study summary is illustrated in Table 3.

#### 4.3.5. PRODIGE-23

PRODIGE-23 is a phase III clinical study whose main goal was to evaluate the efficacy of adding neoadjuvant total chemotherapy to standard radiochemotherapy and determine disease-free survival (DFS); this study included patients with biopsy-proven LARC (cT3/cT4, M0) [9]. Following random allocation, they were divided into two groups: One received standard therapy of chemoradiotherapy (50 Gy, 2 Gy/fr for 5 weeks, concomitant with capecitabine 1600 mg/m^2^, 5 days out of 7), then surgery after 6–8 weeks, and followed by 6 months of ADJCHT, either FOLFOX6 or capecitabine. In the other group, neoadjuvant therapy was employed, consisting of chemotherapy with FOLFIRINOX (oxaliplatin 85 mg/m^2^, irinotecan 180 mg/m^2^, leucovorin 400 mg/m^2^, and fluorouracil 2400 mg/m^2^ every 2 weeks for six cycles), chemoradiotherapy (a total dose of 50 Gy over 5 weeks and concomitant capecitabine 800 mg/m^2^, twice a day, 5 days a week), and surgery—total rectal excision. Moreover, 3 months of modified FOLFOX6 (oxaliplatin 85 mg/m^2^ and leucovorin 400 mg/m^2^, followed by 400 mg/m^2^ fluorouracil bolus and then 2400 mg/m^2^ continuous infusion for 46 h every 2 weeks for six cycles) or capecitabine (1250 mg/m^2^ twice daily on days 1–14, every 21 days) were added to this group. If the 3-year data were encouraging, the 7-year results of this study show the superiority of chemotherapy with FOLFIRINOX compared with preoperative chemoradiotherapy. After 7 years, the disease-free survival (DFS) was 67.6% in the experimental arm versus the standard arm—62.5%. With respect to metastasis-free survival, the FOLFIRINOX group had a rate of 73.6%, while the standard group had 65.4%, also offering superior results for OS (81.9% for the conventional arm versus 76.1% for the experimental arm) [45]. pCR rates were also spectacular in the TNT group, with a complete response in 27.5% of the patients treated with TNT vs. 11.7% in patients treated with standard long-course radiochemotherapy.

The PRODIGE-23 study summary is illustrated in Table 4.

#### 4.3.6. STELLAR

This study evaluates SCRT and neoadjuvant chemotherapy followed by surgery relative to LCRT, surgical resection, and adjuvant chemotherapy. The primary endpoints were DFS and OS [40].

The inclusion criteria were T3/4 locally invasive rectal carcinoma with lymph node extension but without metastases. The study group comprised 599 patients, randomly assigned to either the experimental or standard group.

The approach in the experimental arm comprised SCRT—a total dose hypofractionation of 25 Gy, 5 Gy × 5, followed by four cycles of CAPEOX (oxaliplatin 130 mg/m^2^ on day 1 and capecitabine 1000 mg/m^2^, twice daily, on days 1–14) one week or two weeks post-radiotherapy completion. The standard group underwent long-course radiochemotherapy reaching a total dose of 50 Gy, administered over 5 weeks, 2 Gy/fr. Concurrently, capecitabine 825 mg/m^2^ has been administered twice daily, and as an adjuvant treatment six cycles of CAPEOX have been delivered. In the TNT group, just two cycles of CAPEOX have been dispensed. In the STELLAR study, approximately 25% of each arm did not receive any ADJCHT, meaning that a quarter of each group did not undergo effective systemic therapy.

The 3-year results did not indicate a substantial difference between the two groups in relation to DFS (62.3% in the NCRT group vs. 64.5% in the TNT group), but we note an improvement in the OS in the TNT group, 86.5% vs. 75.1% in the standard therapy arm, along with PRODIGE 23, to obtain these results in terms of OS [40]; this trial obtained a pCR rate of 26.2% in the TNT group versus one of 5.4% in the standard group.

The STELLAR study summary is illustrated in Table 5.

A summary of all the clinical studies regarding TNT is presented in Table 6.

### 4.4. Dose Escalation Strategies for Neoadjuvant Radiation

Radiotherapy was first used in RC in the 1970s [46]. Radiotherapy was initially used in an adjuvant setting in multidisciplinary treatment, which then changed following evidence from the Swedish Rectal Cancer Trial to neoadjuvant settings [30]. The use of hypofractionation in RC is based on the radiobiological properties of rectal adenocarcinoma; however, most major treatment guidelines recommend LCRT with total doses ranging from 45 to 50.4 Gray [24,47,48]. Several studies have proposed increasing the total dose at the tumor level, but the results are controversial. Total doses of up to 54–60 Gray or more have been tried in various trials, administered by external beam radiotherapy with conventional fractionation. A meta-analysis of 487 patients who received radiotherapy doses > 60 Gray summarizes that the complete clinical response (cCR) achievement rate is 20.4% with a resection rate of 89.5% but with a significant grade 3–4 acute toxicity of 10.3% [49]; however, Liang et al. compared survival differences between groups receiving high-dose radiotherapy (above 54 Gy) and those receiving conventional doses and observed no differences [50].

The 2023 French OPERA study analyzed 141 patients and compared two methods of radiotherapy boost administration: either an external 9 Gy in five fractions boost or an X-ray contact brachytherapy boost of 90 Gy in three fractions (also called the Papillon technique). Patients included in the study had tumors up to 5 cm in diameter, were classified as T2 or T3, and received standard neoadjuvant radiochemotherapy with capecitabine and 45 Gy external radiotherapy. The results were particularly encouraging for the group receiving X-ray contact brachytherapy with tumors with a maximum diameter of 3 cm. The organ preservation rate for this group at 3 years was 97%, much higher than the external radiotherapy alone group, which was 63%.

The Papillon technique can also be used for the treatment of small rectal tumors in inoperable patients, with very good results [51]. Steinke et al. published an article in 2023 with 262 patients treated with SCRT and contact brachytherapy. The aim of the study was to avoid surgery in elderly patients (the average age of the study participants was 81 years), 95% of whom remained stoma-free [52]. Other important results worth mentioning are a cCR of 70% in patients who received radiotherapy alone and 97% in those who additionally received local excision, with a 5-year DFS of 53% and 86%, respectively. In the case of friable patients with comorbidities, SCRT with a boost by the Papillon technique is a suitable option.

Depending on the chosen endpoint, we may consider dose escalation of radiotherapy regarding the neoadjuvant treatment of RC. If we are talking about organ preservation, boosting externally or, even better, internally via brachytherapy may increase the chance of cCR and make a patient eligible for the “watch-and-wait” strategy. The big disadvantage of boost with contact brachytherapy is the lack of specialized centers that can perform this technique, with only 14 centers across Europe in 2023 [51]. On the other hand, if we choose overall survival as our endpoint, boosting the dose of radiotherapy does not necessarily bring benefits and can be avoided in order not to cause higher toxicities.

### 4.5. Strategies for Skipping Neoadjuvant Radiotherapy

Historically, there have been several attempts to omit radiotherapy from the neoadjuvant treatment of RC. In 2009 the results of the NCIC-CTG C016 trial were published, giving us an overview of survival of SCRT vs. LCRT provided in an adjuvant setting only in patients with positive circumferential margins. The results were in favor of neoadjuvant radiotherapy in terms of the relative risk of local recurrence, with patients treated with neoadjuvant radiotherapy having a 61% lower chance to relapse locally; survival between the two groups did not differ [53].

Glynne-Jones raised the question of whether we could forego radiotherapy altogether, while retaining chemotherapy, in patients in whom we can thoroughly assess the mesorectal fascia via MRI and in whom we can expect negative postoperative margins. Evidence shows that preoperative radiotherapy reduces local recurrence, but the impact on survival is limited, which may be insufficient to consider radiotherapy necessary [54]. This behavior of radiotherapy is not surprising because radiotherapy is a localized treatment and local control alone does not prevent systemic failure. In addition, preoperative radiotherapy brings additional risks through higher perioperative morbidity [55,56], potentially severe late toxicities [57,58], or secondary radiation-induced neoplasms [59,60].

A feasibility study was proposed in the UK in 2013 and evaluates omitting neoadjuvant radiotherapy in low rectal cancers where negative resection margins are predicted on preoperative MRI [61]. If the study had proved feasible it would have been followed by phase III trials for confirmation. Recruitment for this study is currently indefinitely interrupted. The reasons given were lack of enrolment, too stringent inclusion criteria, and poor organization, although the trial authors believe the research topic is still valid.

Three other retrospective studies evaluated omitting neoadjuvant radiotherapy and using only chemotherapy with FOLFOX +/− bevacizumab [61,62,63,64]. The only outcome that deserves our attention in the previously mentioned study is pCR, which varies between 7% and 35%, while the recurrence or metastasis rate cannot be estimated reliably due to the limited patient population.

The results of the BACCHUS trial, proposed by Glynne-Jones, were published in 2018 [65]. It pursued the idea of neoadjuvant chemotherapy (NACT) without radiotherapy and compared FOLFOX + bevacizumab or FOLFOXIRI + bevacizumab regimens for six cycles versus neoadjuvant LCRT or SCRT. Poor accrual and a high rate of acute adverse reactions (including one suicide) led to poor results, but the authors proposed the further evaluation of the regimen, including irinotecan.

Neither the FOWARC or GEMCAD 0801 trial, which had the same inclusion principles, gave favorable results for omitting neoadjuvant radiotherapy. Patel et al. reported unexpectedly high toxicity through the association of likely bevacizumab-associated adverse reactions [66], while Deng et al. showed in the NACT arm a lower rate of pCR, but also lower toxicity [67].

At the ASCO Annual Meeting in 2023, the first results of the PROSPECT study, a non-inferiority phase II/III study conducted in 264 institutions, were presented [68]. Patient selection was based on age (18 years or older), untreated, pathologically confirmed, and stage T2, with positive lymph nodes or stage T3, with negative lymph nodes or positive lymph nodes; for eligibility, the surgeon’s therapeutic approach had to be neoadjuvant chemoradiotherapy, followed by the preservation of the sphincter. The premise of the study is equivalence between FOLFOX and neoadjuvant chemoradiotherapy for patients with T2N+ or T3 rectal neoplasm in terms of disease-free survival after 58 months of follow-up. The study aims to confirm the non-inferiority of the experimental arm, neoadjuvant FOLFOX with selective LCRCHT in maintaining high pelvic R0 resection rates and time to local recurrence (phase II of the study), and to compare its disease-free survival outcomes to the standard-of-care arm, standard LCRCHT (phase III of the study). In it, 1194 patients were randomized over 6 years and the results for the FOLFOX group were DFS 80.8% (CI95%, 77.9–83.7) and DFS 78.6% (CI95%, 75.4–81.8) for the LCRCHT group, thus achieving the primary objective of the study. The chemotherapy-only arm had nearly double the toxicity rate compared to the radiochemotherapy arm (41% vs. 23%), while overall health-related quality of life was similar across both treatment approaches; however, despite a median follow-up of 58 months, long-term data on toxicity and quality of life have not yet been reported. Some limitations of the study should be mentioned, such as the non-blind randomization, the slightly higher number of T3N+ patients in the LCRCHT group, or the poorer pCR in FOLFOX (21.9 vs. 24.3%). Additionally, a comparison between neoadjuvant FOLFOX and TNT strategies may provide new insights for the future.

Overall, the trial introduces a new treatment option for rectal cancer, highlighting the unique benefits and limitations of both chemotherapy and chemoradiotherapy approaches. Although the trial design may be considered outdated for using LCRCHT instead of TNT as the standard care arm, comparing neoadjuvant FOLFOX with TNT could be of interest for future approaches.

### 4.6. Strategies for Skipping Surgery

The wait-and-watch (WW) method is a safe treatment method that minimizes the risk of surgery, preserving a patient’s immune balance during RC treatment and thus increasing a patient’s tolerance to and compliance with the therapeutic options. The WW approach is chosen for patients with cCR after neoadjuvant therapy and also helps patients with conditions that are not candidates for surgery. Adopting this strategy would simultaneously help reduce the social and personal costs associated with treatment [69]; however, it has been recorded that local recurrence rates are higher when choosing the WW method over radical surgery, but recurrences have a very good radical excision rate. For this reason, constant imaging monitoring of the patient during this strategy is of considerable importance [70].

The WW method indicates a fundamental change in the treatment of RC away from the traditional approach involving NCRT, TME, and postoperative chemotherapy; however, this strategy is only feasible in patients with a complete response to NCRT or TNT [71]. The surgical management of LARC with cCR remains the standard treatment, but uncertainties remain regarding the definition, classification, and safety of delaying surgery in patients with cCR. Given the surgical treatment of RC and the significant surgical risks and reduced QoL, there has been strong interest in the process of WW. It is important to keep in mind that if this method is chosen, the patient is enrolled in a specific follow-up protocol to manage the risk of recurrence. While the follow-up protocol is universally more intense than in other therapeutic strategies, there is currently no standard approach. Byun et al. emphasize the disparities between the follow-up protocols. The OPRA study approach is based on (1) digital rectal examination and sigmoidoscopy every 4 months for 2 years after the initial assessment; (2) then, every 6 months for the next 3 years; (3) rectal MRI every 6 months during the first 2 years; and (4) rectal MRI every 12 months for the next 3 years. Whereas some studies involved digital rectal examination, proctoscopy, and carcinoembryonic antigen testing every 6–10 weeks for the first 2 years of follow-up, others included, besides that mentioned above, a CT scan of the chest, abdomen, and pelvis, and two carcinoembryonic antigen determinations in the first 2 years [72]; therefore, WW has been shown to be an advantageous strategy, but is subject to uncertainty, because no agreement has been achieved on the classification of patients with cCR, the duration of clinical follow-up, the modification of neoadjuvant treatment, and the likelihood of recurrence [73].

The eligibility of the WW strategy is determined via thorough assessment, including physical examination, endoscopy, and imaging. On digital rectal examination, there should be no palpable tumor or ulceration, while endoscopy must confirm the absence of visible tumors, ulcerations, or irregularities. MRI plays a critical role, requiring no residual tumor or suspicious lymph nodes, with normalization of the rectal wall. Negative or inconclusive biopsies may support eligibility but are not always reliable due to fibrosis.

Patients with early stage rectal cancer (cT2N0 or minimally invasive cT3N0) are the most suitable candidates for WW; however, those with locally advanced disease (cT3c/d or cT4) may also be considered if they achieve a robust cCR after neoadjuvant chemoradiotherapy or total neoadjuvant therapy, though the recurrence risk is higher [71]. The approach is particularly beneficial for patients unfit for surgery due to comorbidities or those wishing to avoid the potential morbidity of rectal surgery, such as permanent colostomy. Candidates must commit to a strict follow-up protocol to monitor for recurrence, including regular clinical examinations, endoscopy, and imaging every 3–6 months within the first two years.

Contraindications include residual tumors, incomplete responses on imaging or endoscopy, and evidence of nodal involvement or metastases. Tumors with an aggressive histology or poor differentiation also make WW unsuitable. Long-term surveillance is essential for detecting early regrowth, with intensity decreasing after two to three years. The WW approach offers an effective alternative for well-selected patients, balancing oncologic safety with the preservation of quality of life.

### 4.7. Other Neoadjuvant Therapies

#### 4.7.1. Immunotherapy in Rectal Cancer

Approximately 5–10% of patients with RC have mismatch repair-deficient (dMMR) or microsatellite instability-high (MSI-H) tumor characteristics [3]. For these patients, standard chemotherapy is not optimal, but immunotherapy with immune checkpoint inhibitors (ICIs) using agents targeting programmed death-1 (PD-1) and anti-programmed cell death ligand 1 (PD-L1) is superior.

Cabezon-Gutierrez et al., in 2023, conducted a comprehensive review of studies that evaluated the immunotherapy response in LARC with dMMR or MSI-H [74]. They noted interesting results obtained in the nine remaining studies after sampling and raised some interesting questions about the potential of immunotherapy in RC: Can we omit radiochemotherapy altogether? Can we additionally omit surgery if we achieve a complete response with immunotherapy alone? Which immunotherapy strategy to use? An answer to these questions is offered by the study of Chalabi et al. In their work on neoadjuvant immunotherapy, out of 111 patients 95% had less than 10% residual viable tumors, while 68% achieved a complete pathological response, defined as 0% residual viable tumors [75].

Many other prospective studies, among which we mention only a few (NCT04304209 using sintilimab [76], NCT04165772 with dostarlimab [77], NCT06229041 with TNT and camrelizumab or NCT05176964 with SCRT and tislelizumab [78]) recruit patients, and aim to answer the questions raised by Cabezon-Gurierrez and further evaluate the role of immunotherapy in patients with dMMR or MSI-H. Moreover, a recent promising study by Cerek et al. supports the validation of this approach. In their prospective phase II study on 12 patients (dMMR stage II or III rectal adenocarcinoma), they studied the clinical response and the recurrence. After 6 months of dostarlimab, 100% of patients (95% CI, 74–100) achieved a complete clinical response, maintaining a progression-free and recurrence-free status during the 25-month follow-up [79].

The authors’ conclusion is that it is still too early to decide the optimal treatment for this subcategory of patients, but future studies seem to point to a change in guidelines at least for adding immunotherapy, if not replacing radiochemotherapy altogether. NCCN version 4.2024 published in September 2024 already adds immunotherapy as the first neoadjuvant treatment for MSI-H LARC patients.

#### 4.7.2. Proton Beam Therapy

Proton beam therapy (PBT) is a more complex form of radiotherapy used to treat several types of solid tumors. In RC, PBT may be a treatment option for local recurrence, although it is used much less frequently than photon reirradiation and only in clinical trial settings [80]. The physical properties of proton beams and the use of the Bragg peak to the advantage of healthy organ avoidance bring many theoretical advantages of use in the clinic: higher precision in the tumor, reduced side effects, the protection of organs at risk, and higher therapeutic efficiency compared to photons by using predominantly direct ionization. Unfortunately, all these advantages have not been fully studied clinically in rectal cancers.

Clinical data have been reported for LARC by Japanese groups working with protons on a few dozen patients [81,82,83]. Grade 3–4 adverse reactions are very few, and the 3-year DFS ranges from 44% to 100% for reirradiation using protons. Another small retrospective study was published in 2014 and obtained partial or complete radiographic results in 57% of patients 19 months after re-irradiation [84].

Most studies performed on the proton irradiation of LARC are dosimetric studies that all show a theoretical advantage of protons in preventing acute small bowel, testicular, or bladder toxicities compared to photon irradiation. When talking about photon irradiation techniques, PBT is superior to NCRT and only superior in some aspects to IMRT [85,86].

The PRORECT phase II clinical trial, started in 2021 and conducted at the Karolinska Institute in Sweden, is the first prospective study using PBT for the per primam irradiation of LARC. The study design includes SCRT (5 × 5 Gy regimen) administered with either protons or photons, followed by CAPEOX chemotherapy for a minimum of four series and surgery [87]. The expected outcome is better tolerance to neoadjuvant chemotherapy due to lower acute gastrointestinal toxicity in the PBT arm. The final results of the study are expected in 2028 and may provide a major change in the optimal neoadjuvant radiotherapy regimen.

The trend towards organ preservation strategies could benefit from the dosimetric and clinical advantages of PBT by delivering higher effective biological doses to the tumor, better protection of organs at risk, and higher chances of achieving cCR.

#### 4.7.3. Hyperthermia

Hyperthermia (HT) is a form of complementary cancer treatment that involves heating body tissues, deeply or at the surface, to temperatures between 40 °C and 44 °C for a period of time (usually 1 h). Tumor heating is performed once or twice a week, either right before or right after a radiotherapy or chemotherapy session, ideally just a few minutes apart. The aim of hyperthermia is to improve the efficacy of radiotherapy or chemotherapy by utilizing the benefits it can provide: increased blood flow to the tumor (and therefore increased reoxygenation and reduced hypoxia), the inhibition of DNA repair, direct cell damage and synergistic effects with RT or CHT.

Hyperthermia in RC has recently been studied in several studies and shows promising results: a higher degree of tumor regression in patients in whom a higher temperature was obtained, good oncological outcomes even in high-risk patients, and QoL similar to that of patients not receiving hyperthermia [88,89,90].

Datta et al. report that hyperthermia can alter the alpha–beta ratio of tumor cells, thus altering tumor radiobiological properties and making them more sensitive to fractionation changes [91]. This principle, in conjunction with the fact that the alpha–beta ratio of rectal tumors is controversial [92], could form the basis of a study evaluating the tumor response to the combination of hyperthermia—hypofractionated radiotherapy +/− chemotherapy comparable to the RAPIDO trial but with the addition of HT, a topic that is already under consideration by some hyperthermia working groups.

The development of deep hyperthermia devices (such as ALBA 4D™, MED-LOGIX SRL Rome, Italy) using four antennas to increase the temperature to 43–44 °C in deeply located tumors, such as rectal tumors, opens new horizons in studies to optimize the neoadjuvant treatment of LARC and in the potential for the personalization of treatment, especially due to the advantages of virtually no adverse reactions.

#### 4.7.4. MRI-Guided Radiation Therapy

MRI-guided radiation therapy (MRgRT) is an advanced form of radiotherapy that utilizes magnetic resonance imaging (MRI) to precisely target tumors while minimizing damage to surrounding healthy tissues. This technology offers significant advantages over conventional radiation therapy, particularly in the treatment of rectal cancer. By providing the continuous visualization of tumors and adjacent organs, MRgRT enables radiation oncologists to deliver highly accurate and personalized treatment plans [93]. MRI guidance can be used either offline (not related to treatment sessions, mostly used for planning) or online (intra- and inter-fraction, using MRI-LINAC (Elekta, Stockholm, Sweden), enabling adaptive RT) [94].

Two MRI-LINAC systems are in clinical use: the 0.35 T MRIdian (ViewRay, Inc., Mountain View, CA, USA) and the 1.5 T Unity (Elekta AB, Stockholm, Sweden), each with its unique capabilities. These systems provide high-resolution images of tumors and surrounding tissues, allowing for precise targeting and dose delivery [95].

One of the key benefits of MRgRT in rectal cancer is its ability to account for daily anatomical variations caused by factors such as bowel gas, bladder filling, and tumor movement. By tracking these changes in real time, radiation oncologists can adjust the treatment plan accordingly (adaptive planning), ensuring that the radiation dose is delivered to the tumor with maximum precision [96]. Online adaptive MRgRT, conducted with the patient stationary in the treatment setup, accommodates dynamic anatomical variations, including soft-tissue shifts, volume fluctuations, and the repositioning of nearby organs at risk. Superior visualization of internal anatomy permits shrinking the planning target volume (PTV) margins, thereby minimizing radiation exposure to healthy organs [97]. This increased accuracy can lead to improved tumor control and reduced side effects, such as bowel dysfunction and urinary incontinence.

A significant reduction in doses delivered to organs at risk has already been documented in several studies using MRgRT, with the rate of toxicity being reduced from 10% to 30% [95,98]. However, another major advantage of MRgRT could be the potential for increasing tumor-level radiation doses. A phase I study is already underway, proposing dose escalation to the target through a simultaneous integrated boost, reaching up to 72 Gy [99]. The use of MRgRT in rectal cancer radiotherapy has a bright future and the potential to become the standard approach in rectal cancer irradiation after broader adoption.

In summary, many promising advances in rectal cancer treatment, including immunotherapy, proton beam therapy, hyperthermia, and MRgRT, face significant obstacles. Their high costs and limited access deter broader use, and specialized equipment adds another layer of complexity. While early studies show encouraging results, large-scale trials are still needed to confirm their safety and effectiveness. These barriers must be addressed to fully integrate these innovations into everyday cancer care.

### 4.8. Management of Locally Recurrent Rectal Cancer

The management of locally recurrent rectal cancer (LRRC) hinges on the feasibility of curative treatment, as untreated cases have a poor prognosis, with a median survival of just 6–7 months [100,101]. Palliative approaches, including radiotherapy or chemoradiotherapy, may alleviate symptoms like pelvic pain and prolong survival modestly, but often come with significant side effects. Surgical resection remains the cornerstone for either cure or symptom relief, but it is a complex and challenging procedure due to prior surgical and radiotherapy treatments [102]. Advances in surgical techniques, reconstruction, and multidisciplinary care have significantly improved outcomes, including long-term survival, quality of life, and reduced morbidity [103].

Achieving a microscopically clear surgical margin (R0 resection) is the primary prognostic factor for long-term outcomes [104]. Multidisciplinary team involvement, including specialists in colorectal surgery, oncology, radiology, and reconstructive surgery, is critical for optimizing resection. Tumor location, the extent of local invasion, and patient fitness are key determinants of surgical candidacy. Contraindications include factors such as a poor performance status, extensive pelvic sidewall involvement, and sacral invasion above S2–S3 [104]; however, resectable metastatic disease or isolated pelvic hydronephrosis may not preclude surgery if deemed feasible [105].

The use of neoadjuvant chemoradiotherapy and intraoperative radiotherapy has further increased the rates of R0 resections, leading to improved survival outcomes over the years. Recent data highlight improved surgical outcomes for LRRC, with resection rates rising from approximately 55% to over 70% in more recent cohorts due to advancements in preoperative therapies and surgical techniques [106]. For patients achieving R0 resection, the 5-year overall survival rates have significantly improved, emphasizing the critical role of careful patient selection, multimodal therapy, and evolving surgical expertise in managing this challenging condition [103].

## 5. Conclusions and Future Directions

The aim of this review was to critically appraise the current results of the studies involved in the treatment of RC and to provide insight into new techniques that may evolve in the next years.

A major finding was the promising results regarding TNT treatment. The RAPIDO trial, POLISH 2 study, CAO/ARO/AIO-12 trial, OPRA study, PRODIGE 23 trial, and the STELLAR trial are among the notable investigations in the field of TNT in rectal cancer management, with improved OS ranging from 41% to 86.5%. Additionally, SCRT exhibits high patient compliance and better tolerability, serving as an alternative for patients intolerant to NCRT, while LCRT enhances resection and local control but may increase toxicity. Dose escalation studies demonstrate varied response rates and toxicity profiles. Furthermore, WW strategies, while advantageous, lack consensus. Immunotherapy shows potential, although its optimal integration into treatment protocols remains under investigation. PBT holds promise for organ preservation strategies, offering dosimetric advantages. Additionally, the development of deep hyperthermia devices presents new avenues for enhancing treatment efficacy in deeply located rectal tumors and the use of MRI-weighted radiation therapy increases the quality of precise target irradiation. Future studies should prioritize the development of RCTs that evaluate the overall long-term survival of TNT regiment patients. Furthermore, the incorporation of radio genomics and big data analysis is essential to increase patient-oriented therapy, thereby enabling more tailored and effective treatment strategies.

## Figures and Tables

**Table 1:** Rectal Cancer and Preoperative Induction Therapy Followed by Dedicated Operation (RAPIDO) study summary.

Patient Criteria cT4a/cT4b or EMVI or N2 or MRF or positive lateral lymph nodes	Conventional Arm	Experimental Arm
	LCRT (50/50.4 Gy)	SCRT 5 × 5 Gy CAPEOX/FOLFOX4
Results	DrTF 30.4%	DrTF 23.7%
	pCR 14.3%	pCR 28.4%

cT4a/cT4b (T4a—invasion of peritoneum or peritoneal reflection; T4b—invasion of surrounding organs or structures); EMVI (extramural venous invasion); N2 (≥four suspicious lymph nodes); MRF (mesorectal fascia invasion); LCRT (long-course chemoradiotherapy); SCRT (short-course radiotherapy); CAPEOX (chemotherapy regimen comprising capecitabine and oxaliplatin); FOLFOX4 (chemotherapy regimen comprising oxaliplatin, leucovorin, and 5-FU); DrTF (disease-related treatment failure); and pCR (pathological complete response).

**Table 2:** POLISH II study summary.

Patient Criteria cT3 or cT4 LARC	Conventional Arm	Experimental Arm
	LCRT (50.4 Gy in combination with 5-FU and leucovorin)	SCRT 5 × 5 Gy Three cycles of FOLFOX4
Results	R0 resection 71%	R0 resection 77%

cT3 or cT4 (cT3—tumor extends beyond the muscularis propria into perirectal tissues; cT4—tumor invades nearby organs or structures); LARC (locally advanced rectal cancer); LCRT (long-course chemoradiotherapy); SCRT (short-course radiotherapy); FOLFOX4 (chemotherapy regimen comprising oxaliplatin, leucovorin, and 5-FU); and R0 resection (surgical resection with no residual microscopic disease).

**Table 3:** Organ Preservation of Rectal Adenocarcinoma (OPRA) study summary.

Patient Criteria T3–4, N0 or any T, N1–2	Conventional Arm	Experimental Arm
	NCRT-CNCT	INCT-NCRT
Results		
5-year DFS	71%	69%
5-year TME-free survival	54%	39%

T3-4, N0 (T3—tumor invading through the muscularis propria into perirectal tissues; T4—tumor invading other organs or structures; N0—no regional lymph node involvement); any T, N1-2 (T—any tumor size; N1—1 to 3 positive regional lymph nodes; and N2—4 or more positive regional lymph nodes); NCRT-CNCT (chemoradiotherapy followed by consolidation neoadjuvant chemotherapy); INCT-NCRT (induction chemotherapy followed by chemoradiotherapy); DFS (disease-free survival); and TME-free survival (total mesorectal excision-free survival).

**Table 4:** Neoadjuvant Chemotherapy with FOLFIRINOX and Preoperative Chemoradiotherapy for Patients with LARC (PRODIGE-23) study summary.

Patient Criteria cT3 or cT4 LARC	Conventional Arm	Experimental Arm
		3 months FOLFIRINOX
	NCRT	NCRT
	6 months FOLFOX6/Capecitabine	3 months FOLFOX6/capecitabine
Results		
7-year OS	81.9%	76.1%
MFS	73.6%	65.4%
pCR	11.7%	27.5%

cT3 or cT4 LARC (cT3—tumor extends beyond the muscularis propria into perirectal tissues; cT4—tumor invades nearby organs or structures; and LARC—locally advanced rectal cancer); NCRT (chemoradiotherapy); FOLFOX6 (chemotherapy regimen comprising oxaliplatin, leucovorin, and 5-FU); FOLFIRINOX (chemotherapy regimen comprising oxaliplatin, leucovorin, irinotecan, and 5-FU); OS (overall survival); MFS (metastasis-free survival); and pCR (pathological complete response).

**Table 5:** STELLAR study summary.

Patient Criteria cT3 or cT4 LARC	Conventional Arm	Experimental Arm
	LCRT	SCRT
	Capecitabine 825 mg/m^2^ ×2/day	Six cycles of CAPEOX
	Six cycles of adjuvant CAPEOX	Two cycles of adjuvant CAPEOX
Results		
3-year OS	75.1%	86.5%
pCR	5.4%	26.2%

cT3 or cT4 (cT3—tumor extends beyond the muscularis propria into perirectal tissues; cT4—tumor invades nearby organs or structures); LARC (locally advanced rectal cancer); LCRT (long-course chemoradiotherapy); CAPEOX (chemotherapy regimen comprising capecitabine and oxaliplatin); SCRT (short-course radiotherapy); OS (overall survival); and pCR (pathological complete response).

**Table 6:** Total neoadjuvant therapy clinical trials—fact comparison.

Study	Conventional Approach	Total Dose (Gy); fr#	1. Primary Endpoints 2. Secondary Endpoints	Study Design and Period	Number of Patients	Clinical Stage	Toxicities	Results
	TNT Approach							
POLISH II	NCRT→TME	50.4 Gy /28#	1. R0 resection 2. Distant metastasis; local failures; OS; PFS; early and late toxicity; and complete pathological response	RCT; 2008–2012	254	cT3—171 cT4—329 Recurrent—16	Third grade—16 Fourth grade—5	OS: 49% (both arms) DFS: 43%
	SCRT + FOLFOX4→TME	25 Gy /5#			261		Third grade—19 Fourth grade—4	DFS: 41%
PRODIGE-23	NCRT→TME→FOLFOX/CAPE	50 Gy /25#	1. DFS 2. OS	RCT; 2012–2019	230	cT2 —5 cT3 —379 cT4 —77	No data	DFS: 62.5% OS: 76.1%
	FOLFIRINOX→ NCRT→TME→CHT	50 Gy /25#			231			DFS: 67.6% OS: 81.9%
STELLAR	NCRT→ TME +/− CAPEOX	50 Gy /25#	1. DFS 2. OS	RCT; 2015–2023	297	cT2 —16 cT3 —497 cT4 —86	Third/fourth grade—13	DFS: 62.3% OS: 75.1%
	SCRT→CAPEOX→TME +/− CAPEOX	25 Gy /5#			302		Third/fourth grade—26	DFS: 64.5% OS: 86.5%
RAPIDO	NCRT→TME→FOLFOX4/CAPEOX	50 Gy /25# or 50.4 /28#	1. DrTF 2. OS; CRM negative rate; pCR rate; early and long-term toxicities; surgical complications	RCT; 2011–2020	450	cT2/3N0—43 cT2/3N+—567 cT4N0 —46 cT4N+ —245	Third grade—23, Fourth grade—2	DrTF: 23.7%
	SCRT→FOLFOX4/CAPEOX→ TME	25 Gy /5#			462		Third grade—41 Fourth grade—7	DFS: 30.4%

TNT (total neoadjuvant therapy); NCRT (neoadjuvant chemoradiotherapy); TME (total mesorectal excision); OS (overall survival); PFS (progression-free survival); DFS (disease-free survival); RCT (randomized controlled trial); SCRT (short-course radiotherapy); FOLFOX (chemotherapy regimen comprising oxaliplatin, leucovorin, and 5-FU); CAPEOX (chemotherapy regimen comprising capecitabine and oxaliplatin); CHT (chemotherapy); DrTF (distant treatment failure); CRM (circumferential resection margin); pCR (pathological complete response); Rectal Cancer and Preoperative Induction Therapy Followed by Dedicated Operation (RAPIDO); and Neoadjuvant Chemotherapy with FOLFIRINOX and Preoperative Chemoradiotherapy for Patients with LARC (PRODIGE-23); #: fractions of the total dose of radiation

## Data Availability

Data are contained within the article.

## Abbreviations

The following abbreviations are used in this manuscript:

MDPI	Multidisciplinary Digital Publishing Institute.
DOAJ	Directory of open access journals.
TLA	Three-letter acronym.
LD	Linear dichroism.
ADJCHT	Adjuvant chemotherapy.
CAO/ARO/AIO-12	Chemoradiotherapy Plus Induction or Consolidation Chemotherapy as Total Neoadjuvant Therapy.
CAPEOX	Chemotherapy regimen comprising capecitabine and oxaliplatin.
cCR	Clinical complete response.
CRC	Colorectal cancer.
CRM	Circumferential resection margin.
DFS	Disease-free survival.
DrTF	Disease-related treatment failure.
EMVI	Extramural venous invasion.
FAK	Focal adhesion kinase.
FOLFOX	Chemotherapy regimen comprising oxaliplatin, leucovorin, and 5-FU.
FOLFIRINOX	Chemotherapy regimen comprising oxaliplatin, leucovorin, irinotecan, and 5-FU.
INCT-CRT	Induction chemotherapy followed by chemoradiotherapy.
LARC	Locally advanced rectal cancer.
LCRT	Long-course chemoradiotherapy.
MFS	Metastasis-free survival.
MMR	Mismatch repair.
MRI	Magnetic resonance imaging.
MRF	Mesorectal fascia.
MRgRT	MRI-guided radiation therapy.
NCT	Neoadjuvant chemotherapy.
NCRT	Neoadjuvant chemoradiotherapy.
OPRA	Organ Preservation of Rectal Adenocarcinoma.
OS	Overall survival.
pCR	Pathological complete response.
PRODIGE-23	Neoadjuvant Chemotherapy with FOLFIRINOX and Preoperative Chemoradiotherapy for Patients with LARC.
QoL	Quality of life.
RAPIDO	Rectal Cancer and Preoperative Induction Therapy Followed by Dedicated Operation.
RCT	Randomized controlled trial.
RC	Rectal cancer.
SCRT	Short-course radiotherapy.
TME	Total mesorectal excision.
TNT	Total neoadjuvant therapy.
WW	Watch-and-wait.
